# Clinical and Microbiological Characteristics of Infective Endocarditis at a Cardiac Center in Saudi Arabia

**DOI:** 10.1007/s44197-021-00013-5

**Published:** 2021-11-04

**Authors:** Mazin Barry, Syed Abdul Bari, Muhammad Yasin Akhtar, Faizah Al Nahdi, Richilda Erlandez, Abdullah Al Khushail, Yahya Al Hebaishi

**Affiliations:** 1 Division of Infectious Diseases, Department of Internal Medicine, College of Medicine, King Saud University, PO Box 2925, Riyadh, 11461 Saudi Arabia; 2 Department of Infection Control, Prince Sultan Cardiac Center, Riyadh, Saudi Arabia; 3 Department of Adult Cardiology, Prince Sultan Cardiac Center, Riyadh, Saudi Arabia

**Keywords:** Infective endocarditis, Rheumatic heart disease, Congenital heart disease, *Staphylococcus*

## Abstract

**Background:**

Infective endocarditis (IE) is a serious disease with complex pathology and significant mortality. Little information is known regarding clinical and microbiological characteristics in Saudi Arabia. This study surveyed these characteristics at a Cardiac Center in Riyadh, Saudi Arabia over a period of 5 years.

**Methods:**

This retrospective study was done on all infective endocarditis (IE) patients admitted to Prince Sultan Cardiac Center between January 1, 2015, and December 31, 2019. Clinical characteristics, microbiological results, management, and outcomes were assessed.

**Result:**

A total of 340 cases of infective endocarditis were identified over the study period.

Most patients (64%) were 50 years old or above, and 67% were males. Fever was the most common clinical presentation, and a murmur was audible in a fifth of patients. Blood cultures were positive in 177 (52%) cases. The most common organisms were *Staphylococcus aureus*, coagulase negative *Staphylococcus* and viridans group *Streptococcus.* Most common microbiological organisms causing native valve endocarditis were viridans group *Streptococcus* (32%) followed by methicillin-susceptible *Staphylococcus aureus* (21%), and for prosthetic valve endocarditis they were coagulase negative *Staphylococcus* (32%) followed by methicillin-susceptible *Staphylococcus aureus* (23%), the most common causes of culture negative endocarditis were Q-fever and brucellosis. Predisposing cardiac conditions were present in 127 (37%) patients, most commonly rheumatic heart disease and congenital heart disease. Surgical intervention was done in 26% of cases, with an overall in-hospital mortality rate of 6.76%.

**Conclusion:**

We demonstrate the epidemiological, clinical, and microbiological profile of infective endocarditis in a tertiary care cardiac center in Saudi Arabia. It gives information concerning the prevalence of responsible organisms. This information will be helpful in assessing patients with suspected IE and in planning management of cases knowing the relative frequency of types of microorganisms encountered.

## Introduction

Infective endocarditis (IE) remains a potentially lethal disease with varied clinical presentations and significantly changing epidemiology. It may be classified as acute, sub-acute, or chronic depending on the course of infection, however, it is more commonly classified according to the type of valve involved into native or prosthetic valve endocarditis which influences the causative pathogens. Recent reports from developed countries have focused on changes in the epidemiology, microbiology, and clinical features of IE [1] as well as major advances in diagnosis and management of this clinical disease [1, 2]. The incidence of IE is estimated to be 0.16–5.4 cases per 1000 hospital admissions. Most patients are aged between 30 and 60 years old with a male predominance [1].

Since Osler’s first description in 1885, the clinical features of IE have changed due to decrease in rheumatic heart disease, an increasing percentage of elderly people, comorbidities, nosocomial exposures, prosthetic valves, intra-cardiac devices, intravenous drug use, and hemodialysis [3]. A microbiological shift from *streptococci* spp. to *staphylococci* spp. as the more frequent causative pathogen has been significantly noted [4]. With increasing rates of multidrug resistant organisms including Methicillin-resistant *Staphylococcus aureus* [5]. Due to disease complexity, the diagnosis of IE is standardized by the Duke-Li classification which combines two major criteria related to microbiology and imaging with five minor criteria. In cases of prosthetic valve IE the use of radiolabeled leukocyte scintigraphy or positron emission tomography (PET) can further enhance the diagnosis [6], in addition to the use of polymerase chain reaction (PCR) assays for blood culture-negative IE [7]. Despite the disease’s well described clinical features, diagnosis and treatment can still be challenging with an overall mortality reaching up to 20%, as IE is a complex disease which may vary depending on the first organ involved, underlying cardiac disease, causative microorganism, presence or absence of complications, and the patient’s underlying characteristics [5].

Data regarding clinical and microbiological features of IE in the Kingdom of Saudi Arabia (KSA) are limited, as one report estimated the rate of definitive IE in KSA to be about 15 cases per 100,000 admissions/discharges [8], while internationally the estimated incidence is 3–10 cases per 100,000 per year [9]. The aim of this retrospective case series was to describe the clinical, microbiological and echocardiographic characteristics of patients with IE over a five-year period at Prince Sultan Cardiac Center (PSCC), a governmental hospital in the Central region of KSA, located in the capital city Riyadh. The hospital is comprised of 220 hospital beds and is the largest cardiac center in the country, with comprehensive cardiac services, serving referred patients from all over KSA.

## Materials and Methods

### Study Design

A systematic retrospective review of medical records of all patients admitted to PSCC from January 1, 2015, to December 31, 2019, with the diagnosis of definite infective endocarditis according to the modified Duke criteria was included.

The study was reviewed and approved by the center’s ethics committee board with research file number R20012.

### Demographic and Clinical Data

Data from medical records were collected on the following: demographic variables: age, gender, clinical presentation data including symptoms and signs were noted. Transthoracic and transesophageal echocardiography were performed, and data collected regarding, type of valve, type of underlying cardiac abnormalities and any other abnormalities. Rate of surgical intervention as well as variable complications including congestive heart failure, systemic embolization, stroke, and renal impairment were reviewed and recorded.

### Microbiological Data

Data were collected on all blood culture results, type of microorganism and antibiotics susceptibility. The microbiology laboratory used the standardized methods to identify microorganisms and the antimicrobial susceptibility. Blood culture-negative endocarditis had additional testing which include fungal cultures, serology for *Coxiella burnetti* using enzyme Immunoassay serology by Bioscientia® Institute of medical diagnostics Germany and Indirect Immunofluorescence test by Mayo® clinic USA, as well as *Brucella melitensis*, *Brucella abortus* and *Bartonella hensale* serologies by enzyme linked immunoassay.

Statistical analysis by descriptive statistics: (using means and standard deviations for continuous variables and frequencies for qualitative variables) using SPSS software package.

## Results

A total of 345 patients were admitted to PSCC with the diagnosis of infective endocarditis, of which 340 fulfilled modified Duke’s Criteria, of those 228 cases (67%) were male and 112 cases (33%) were female. Most patients were between 51 and 60 years with a range of 18–99 years. Mean age was 48 years. Age distribution is listed in (Table 1).

**Table 1:** Age and gender distribution

Age (years)	No. of patients (*N* = 340)	%
18–20	34	10
21–30	43	12.64
31–40	47	13.82
41–50	60	17.64
51–60	61	17.94
61–70	54	15.88
> 70	41	12.05
Mean ± SD of the Age group 48.09 ± 18.94		

Gender	No. of the patients (*N* = 340)	%
Male	228	67.05
Female	112	32.94

*Fisher exact test

*P* = 0.005

### Clinical Manifestations

Fever was the most common clinical finding in 320 (94%) of patients, fatigue and sweating in 58 (17%) patients, dyspnea was present in 14 patients (4.11%), an audible murmur was present in 61 cases (18%). Clinical findings are listed in (Table 2).

**Table 2:** Clinical findings and risk factors of IE patients

Symptoms/Signs	No. of patients	%
Fever with murmur	61	18
Shortness of Breath	14	4.1
Stroke	04	1.2
Splenomegaly	05	1.5
Hepatosplenomegaly	14	4.1
Others—DVT	01	–
Prosthetic valve	109	32
Rheumatic heart disease	51	15
Congenital heart disease	32	9.4
Intra-cardiac device	28	8.2
Hemodialysis line	12	3.5
Nosocomial	4	1.1
Dental procedures	7	2.0

Deep vein thrombosis

Fisher exact test

*P* < 0.005

### Predisposing Factors

Risk factors and underlying heart disease in patients with IE are shown in (Table 2). Of the 340 patients, 109 (32%) had prosthetic valve endocarditis and 111 (32.6%) patients had native valve endocarditis. 51(15%) patients had rheumatic heart disease with valvular lesions and 32 (9.4%) patients had congenital heart disease (5 cases with ventricular septal defect, 10 cases with Tetralogy of Fallot, 1 case with atrial septal defect).

### Microbiology

Blood cultures were incubated in BD BACTEC™ system, identification and susceptibility were performed using VITEK® 2 microbial identification system. A total of 177 patients (52.05%) had positive blood cultures (Table 3). The most common isolated organisms were *Staphylococcus* spp. which was found in 80 patients of which 53 were due to *Staphylococcus aureus* with 36 being methicillin-susceptible *Staphylococcus aureus* (MSSA) and 17 methicillin-resistant *Staphylococcus aureus* (MRSA), while 27 were due to coagulase-negative *Staphylococcus*. The second most common isolated organisms were *Streptococcus spp.* In 37 patients, *Enterococcus* spp. in 27 patients. For blood culture-negative endocarditis, the most common cause was Q-fever in 19 of the 32 patients with congenital heart disease, followed by brucellosis, these and other microbiological etiological agents are outlined in (Table 4).

**Table 3:** Total number of blood cultures (*N* = 340)

Culture	Number	%
Culture positive	177	52

Bacterial culture positive No = 172 (97.1%)		
Gram positive bacteria	144	83.72
Gram negative bacteria	28	16.27
Fungal (*Candida* Spp.)	05	2.82

*Fisher exact test

*P* < 0.005

**Table 4:** Microbiological agents

Gram positive organisms (No = 144)			
Organism	*Staphylococcus* spp. 80 (55.5%)	*Enterococcus* spp. 27 (18.75%)	*Streptococcus* spp. 37 (25.%)
	Methicillin-susceptible *S. aureus* (MSSA)- 36(45%)	*Enterococcus faecalis*- 22 (81.5%)	*Streptococcus. viridans* 29 (78.4%)
	Methicillin resistant *S. aureus* (MRSA)-17(21%)	Vancomycin resistant *Enterococcus* (VRE)- 5 (18.5%)	*Streptococcus Salivarus* 2 (5%)
	Coagulase negative *Staphylococcus* (CONS)-27(34%)		*Streptococcus milleri* 2 (5.4%)
			*Streptococcus bovis* 1 (3%)
			*Streptococcus. angiosus* 1 (3%)

Gram negative organisms (No = 28)		
Name of the organism	No. of isolates & %	Drug resistance
*Klebsiella pneumoniae*	9 (32.1%)	MDR 5 (55.5%) XDR 1 (11.1%)
*Enterobacter cloacae*	2 (7.1%)	–
*Burkholderia cepacia*	1 (3.5%)	–
*Stenotrophomonas maltophilia*	1 (3.5%)	–
*Ochrobacterium anthropi*	1 (3.5%)	–
*Aggregatibacter aphrophilus*	1 (3.5%)	–
*Brucella melitensis*	12 (42.8%)	–
*Niesseria elongate*	1 (3.5%)	

Culture negative endocarditis (Serology Positive)		
Organism	Positive (*N* = 30)	%
*Coxiella burnetii*	19	63.3
*Brucella* spp.	9	30
*Chlamydia pneumoniae*	1	3.3
*Bartonella henselae*	1	3.3

*MDR* multidrug resistant, *XDR* extensively drug resistant

*Fisher Exact test

*P* < 0.005

Type of positive isolates in native and prosthetic valves are shown in (Table 5). Of the Total 177 isolates from 340 patients, 172 were bacterial and 5 were fungal isolates, out of which 158 (89.27%) were positive in combined native and prosthetic valves infective endocarditis, the remaining 19 (10.73%) isolates were device or health care associated.

**Table 5:** Total positive isolates in NATIVE and prosthetic valves-158 (89.2%)

Native valve endocarditis	111 (32.6%)	Prosthetic valve endocarditis	109 (32%)
Bacterial isolate in native valve endocarditis		Bacterial isolate in prosthetic valve endocarditis	
Total isolate	70 (44.3%)	Total isolate	88 (55.7%)
Bacterial	67 (95.7%)	Bacterial	87 (98.8%)
Fungal	3 (4.3%)	Fungal	1 (1.2%)

Gram positive organisms	57 (85%)	Gram positive organisms	75 (86.2%)
*Staphylococcus* Species(26) 38.8%		*Staphylococcus* Species (54) 62%	
MSSA	16 (23.8%)	MSSA	19 (21.8%)
CONS	5 (7.5%)	CONS	28 (32.1%)
MRSA	5 (7.5%)	MRSA	7 (8%)
*Streptococcus viridans*	18 (31.5%)	*Streptococcus viridans*	9 (10.3%)
*Enterococcus faecalis*	11 (19.30%)	*Enterococcus faecalis*	8 (9.1%)
*Streptococcus anginosus*	1 (1.75%)	*Streptococcus salivarus*	1 (1.14%)
*Streptococcus hominis*	1 (1.75%)	*Streptococcus agalactiae*	1 (1.14%)
–		*Streptococcus mitis*	1 (1.14%)
–		*Streptococcus bovis*	1 (1.14%)

Gram negative organisms	10 (15%)	Gram negative organisms	12 (13.8%)
*Klebsiella pneumoniae*	6 (60%)	*Klebsiella pneumoniae*	4 (33.3%)
*Brucella melitensis*	3 (30%)	*Brucella melitensis*	3 (25%)
*Ochrobacterium anthropic*	1 (10%)	*Enterobacter cloacae*	2 (16.6%)
		*Aggregatibacter aphrophilus*	1 (8.3%)
		*Burkholderia cepacia*	1 (8.3%)
		*Nisseria elongatata*	1 (8.3%)

Fungal		Fungal	
*Candida albicans*	3 (4.3%)	*Candida albicans*	1 (1.1%)

*MSSA* Methicillin-susceptible *Staphylococcus aureus,* *MRSA* methicillin-resistant *Staphylococcus aureus,* *CONS* coagulase-negative *Staphylococcus*

Fisher exact test

*P* < 0.005*

### Echocardiography

Echocardiography was performed in all patients by transthoracic echocardiography (TTE), transesophageal echocardiography (TEE) was done in 224 (65.8%) patients.

The average vegetations size was + 7.5 mm. Vegetations on the mitral valve were detected in 122 (36%) patients, of those moderate to severe mitral regurgitation was detected in 101 (82%) patients. The aortic valve was involved in 79 (23%) patients, of the latter 51 (64%) had moderate to severe regurgitation. Severe tricuspid regurgitation in 17 patients (5%), the pulmonary valve in 14 patients (4%). Mitral and aortic valve were both affected in 24 patients (7%), Mitral and tricuspid valves in 13 (3.8%) patients, and pulmonary with tricuspid valves in 1 patient.

### Treatment, Complications, and Outcome

All patients received at least two intravenous antibiotics according to culture results, ceftriaxone was the most common antimicrobial used (62%), followed by vancomycin (56%), cefazolin (29%), and gentamicin (18%). In cases of culture-negative endocarditis, empirical antibiotic therapy was applied.

Complications that required surgical intervention occurred in 89 patients (26.17%); the indications for surgery were congestive heart failure in 30 patients (9%), mobile vegetation larger than 1.2 cm in 26 patients (7.6%), systemic embolization in 18 patients (5%), stroke in 15 patients (2.2%). Renal failure requiring renal replacement therapy occurred in 67 patients (20%) The in-hospital mortality rate attributed directly to IE was 6.76% (23 patients).

## Discussion

Infective endocarditis is still associated with high mortality and complications. Changes have been reported in the epidemiological and microbiological profile of IE over the last 30 years [10]. IE is more commonly seen in older age groups with a predominance of male patients and an increased incidence of acute IE caused by virulent organisms such as *Staphylococcus aureus*, while Gram-negative organisms are seen in nosocomial and device related infections.

In common with reports from developed countries, our study reveals that the male to female ratio agreed with most case series [11]. While our patient population had a mean age of 48 years, 55% of patients were aged above 50 years and 12% of patients were aged above 70 years. In comparison, a study from a hospital in the Eastern region of KSA by Al-Tawfiq et al. on 83 IE patients over a thirteen-year period reported a mean age of 59.7 years [8], while another study from Aseer region by Assiri on 44 patients over a five-year period reported a mean age of 31 years [12]. A study by Zaqout et al. from Qatar, a neighboring country to KSA, examined 57 patients with IE over a three-year period, they reported the mean age to be 51 years [13]. Elsewhere data from the United States has shown that more than 50% of all IE cases were seen in patients over the age of 60 years, with a steady increase in the mean age of 45.3 years in the 1980s to 57.2 years in the 2000s [14]. This trend in developed countries is probably due to two factors: the increasing proportion of elderly people in the general population and the decline in the incidence of rheumatic heart disease [15]. In our patient population, second to prosthetic heart valves, rheumatic heart disease was the most common underlying cardiac condition as reported by Andrea et al. [16].

Fever was found to be the most common symptom in our cohort which was reported in more than 90% of patients which is similar to that reported by Assiri [12], as well as to that reported in most patients presenting to the emergency department with IE [17], however, fever was reported slightly less by Zaqout et al. in Qatar (84%). Only one-fifth of our patients had a documented audible murmur which is much lower than a study by Yakut et al. that reported a new murmur or changing murmur in 68% of patients [18]. Previous studies from KSA and Qatar did not report the number of murmurs in their cases. Splenomegaly with or without hepatomegaly was documented in 5.5% of our patients, detecting splenomegaly during assessment of patients with suspected IE is important, Rohani et al. reported a case of IE presenting with isolated splenomegaly [19]. Systemic embolization and/or stroke was observed in almost 7% of our patients, which is similar to that reported by Zaqout et al., while Assiri reported stroke in 9% of patients and another 4.5% developed peripheral septic emboli. A study from Spain reported embolic events in almost 12%, mostly affecting the central nervous system [20]. Sixty-seven or 20% of our study population developed acute kidney injury that required renal replacement therapy, the study from Qatar reported acute kidney injury in 30%, while that from Aseer reported renal failure in 4.5%, both studies did not report the requirement of renal replacement therapy. A French hospital discharge database on 112 patients with IE showed that 27% developed stage 3 acute kidney injury, while only three patients required renal replacement therapy [21].

In certain clinical situations, a combined medical and surgical approach is necessary for the successful treatment of IE. During the last three decades, valve replacement and repair have become common practice in the management of selected complications of IE, and the combination of antibiotic therapy and timely surgical intervention has substantially reduced the mortality from IE. Our results demonstrate that 26% of IE cases required surgical intervention, which, like other reports, showed it to be between 25 and 50% during acute infection and 20–40% during convalescence [22–24]. The previous report from Aseer showed that 50% of patients underwent surgery [12], while only 16% of those in Qatar required surgical intervention [13]. Over time, the indications for surgery have been extended and valve replacement surgery has been undertaken progressively earlier in the course of the illness. The indications for surgery in our patients were consistent with the guidelines of the American College of Cardiology/American Heart Association, namely congestive heart failure, systemic embolic events, valve dysfunction, failure of medical therapy and perivalvular complications such as abscess formation and large mobile vegetation.

After prosthetic valves, rheumatic heart disease was the most common risk factor of IE in our study, Assiri also reported it to be the highest risk factor among 44 patients [12]. The third most common predisposing factor for IE was congenital heart disease reported in almost one-tenth of our study population, which is much higher than that reported in Qatar [13]. Interestingly, *Coxiella burnetii*, the causative agent of Q-fever, was common in in our congenital heart disease cohort, this can be explained in part by cardiac implants, bovine jugular transplants and the endemicity of Q-fever in some regions of Saudi Arabia [25].

The number of native valve IE and prosthetic valve IE were 111 (32.6%) and 109 (32%), respectively, which is nearly equal, one explanation would be the referral of complicated prosthetic valve cases from other hospitals around the whole Kingdom, causing higher reports from developed countries for prosthetic valve IE, the previous report from the Eastern region showed that only 18.5% were prosthetic valve IE [8], and the study from Aseer region reported it to be 22.7% [12], while the study from Qatar reported it to be 19% [13].

The yield of microorganisms isolated from blood cultures (52.05%) was closer to a study by Ghosh from North India [26]. Low yield could be due to prior use of antibiotics before cultures were withdrawn and referred patients may have already been on treatment that was started in the original referring hospitals. *Staphylococcus* spp endocarditis was predominantly reported in this study which agrees with several reports from developed countries [27], as we found that MSSA caused 24 and 22% of culture-positive native and prosthetic valve endocarditis, respectively, while MRSA caused almost 8% of both native and prosthetic valve IE. Overall, the most frequent isolate in prosthetic valve IE was coagulase negative *staphylococcus* (CONS) (32.1%), Al-Tawfiq et al. similarly reported *staphylococcus* spp as the most common microorganism with *staphylococcus aureus* in nearly 40%, MRSA in 4%, CONS in 7.4% [8], while Assiri reported *staphylococcus aureus* in 18.2% and CONS in 4.5%. In Qatar, MSSA, MRSA, CONS were reported to be 14, 11, 9%, respectively. In our study Viridans group streptococci caused 31.5 and 10.3% of native and prosthetic valve IE, respectively, both Assiri and Zaquot et al. reported it to cause 14% [12, 13], while Al-Tawfiq et al. reported it to cause 17% [8], none of these studies segregated their microbiology data into native or prosthetic valve IE.

Antimicrobial resistance was noticeable in 28 bacterial isolates (16.3%) out of which 22 (78.5%) were gram positive organisms and 6 (21.4%) were gram negative organisms, In Eastern region 9% were gram negative bacteria, of which one case was due to ESBL-producing *klebsiella pneumoniae* [8], similarly we report five and one cases of MDR and XDR *klebsiella pneumoniae*, respectively.

Over the last 20 years echocardiography has complimentary roles in the diagnosis and evaluation of endocarditis. The sensitivity of TTE in this study was 63.6% which was lower than that reported by Al-Tawfiq et al. at 72% [8], the low negative predictive value confirms that TEE would be essential to diagnose IE when TTE is Inconclusive [28].

A large body of evidence has shown that aortic valves have replaced mitral valves as the most common infected site in IE. However, we observed a predominance of mitral valve infection [29]. Similarly, all previous studies from the region reported predominance of mitral valve involvement [8, 12, 13]. Our data also indicates that 27% of patients had complications which have been previously reported [22]. In-hospital mortality was 6.76%, which is lower than what is reported in most case series of 24% [30]. The mortality rate of our cases due to *Staphylococcus aureus* endocarditis 11(47.82%) is inconsistent with other reports with mortalities of 25% [31, 32]. Numerous studies had previously reported a wide range of in-hospital mortality between 15 and 31% [30, 33–39], in Qatar the mortality was 25% [13], and the study from Eastern KSA reported a mortality rate of 29.4% [8].

Although the present study evaluates a large number of IE cases over a five-year period with scarce studies in KSA [12], it has many limitations including it focusing on a single cardiac center with possible influence of referral bias, it is a retrospective study with few missing data including treatment duration, as well as long term follow-up, however, this is the first study from a major cardiac center (PSCC) that serves as a referral center for all KSA, hence, the descriptive epidemiological data may reflect most cases within the country, in addition, this is the largest case series of IE reported in the region, with various predisposing factors including rheumatic and congenital heart diseases, and the most diverse detailed microbiology data from KSA.

## Conclusion

Over a five-year period in the largest cardiac center in KSA, 340 patients were diagnosed with infective endocarditis, fever was the most common presenting symptom, one-third of all cases had prosthetic valve endocarditis, and rheumatic and congenital heart diseases were the most common predisposing factors. A wide variety of causative microorganisms were identified with majority of cases caused by staphylococci spp. and streptococci spp. while MDR and XDR gram-negative bacteria were rare. Culture negative endocarditis was mostly caused by Q-fever and brucellosis. Surgical intervention was done in one-quarter of all patients. The overall in-hospital mortality was low. Prospective multicenter studies in KSA to validate and expand on these results are warranted.

## Data Availability

All data are available upon reasonable request to corresponding author.

## Abbreviations

IE: Infective endocarditis
KSA: Kingdom of Saudi Arabia
PSCC: Prince Sultan Cardiac Center
CBC: Complete blood count
DVT: Deep vein thrombosis
MSSA: Methicillin Sensitive *Staphylococcus Aureus*
MRSA: Methicillin resistant *Staphylococcus Aureus*
CONS: Coagulase negative *Staphylococcus*
TTE: Transthoracic echocardiography
TEE: Transesophageal echocardiography
VRE: Vancomycin resistant *Enterococcus*
MDR: Multidrug resistant organism
XDR: Extensively drug resistant organism
